# Computational Prediction of Heme-Binding Residues by Exploiting Residue Interaction Network

**DOI:** 10.1371/journal.pone.0025560

**Published:** 2011-10-03

**Authors:** Rong Liu, Jianjun Hu

**Affiliations:** Department of Computer Science and Engineering, University of South Carolina, Columbia, South Carolina, United States of America; University of South Florida College of Medicine, United States of America

## Abstract

Computational identification of heme-binding residues is beneficial for predicting and designing novel heme proteins. Here we proposed a novel method for heme-binding residue prediction by exploiting topological properties of these residues in the residue interaction networks derived from three-dimensional structures. Comprehensive analysis showed that key residues located in heme-binding regions are generally associated with the nodes with higher degree, closeness and betweenness, but lower clustering coefficient in the network. HemeNet, a support vector machine (SVM) based predictor, was developed to identify heme-binding residues by combining topological features with existing sequence and structural features. The results showed that incorporation of network-based features significantly improved the prediction performance. We also compared the residue interaction networks of heme proteins before and after heme binding and found that the topological features can well characterize the heme-binding sites of apo structures as well as those of holo structures, which led to reliable performance improvement as we applied HemeNet to predicting the binding residues of proteins in the heme-free state. HemeNet web server is freely accessible at http://mleg.cse.sc.edu/hemeNet/.

## Introduction

Heme proteins, a group of proteins containing an iron–porphyrin complex as a prosthetic group, are found in all living organisms [1]. These proteins carry out a wide variety of basic functions essential for the survival of organisms, such as electron transfer, catalysis, oxygen transport and storage, ligand binding, signal transduction, and gene expression [2], [3], [4], [5], [6], [7], [8]. Due to the diversity of their functions, heme proteins have been the central scientific interest of a great deal of work over the past half century, in which the application of different experimental techniques plays an irreplaceable role in exploring the nature of these biologically important proteins. Although we have gained large amounts of general knowledge about the interactions between heme and its host proteins, the intensive labor and high cost remain the major limitation for experimental techniques. As a consequence, it is necessary to develop effective computational schemes that can assist experimental methods in elucidating the mechanism of heme–protein interactions.

As is well known, the most common types of hemes in nature are heme *b* and heme *c*. Heme *b* binds non-covalently to the protein, whereas heme *c* differs from heme *b* in that the heme vinyl groups covalently contact with two cysteine residues. With the progress of structural genomics, an increasing number of protein structures carrying heme *b* and heme *c* are deposited into the Protein Data Bank [9], which makes it possible to conduct structural and functional studies on heme proteins using computational approaches. Recently, some efforts have been made in this field by several research groups. By means of structural superposition, Schneider et al. [10] first demonstrated that *b*-type heme proteins with different folding topologies are possible for binding the chemically identical heme ligand. They also found that key residues shared by distinct proteins can define some common structural heme-binding motifs, despite considerable diversity existing in heme–protein interactions. Fufezan et al. [11] analyzed the geometric properties of heme-binding motifs and conducted electrostatic and molecular mechanics calculations based on *b*- and *c*-type heme proteins. They proposed that the *b*- and *c*- type hemes have different propensities for different ligation motifs and for the orientations of the histidine heme ligands relative to the heme plane. Subsequently, Smith et al. [12] performed a comprehensive analysis on a dataset of non-homologous heme proteins, and further concluded the characteristics of the binding pockets that recognize and bind heme ligands as well as the features that enable heme groups to perform different biological functions. More recently, Li et al. [13] investigated the differences between the apo and holo structures of heme proteins according to their global structures and binding pockets. It was found that heme proteins generally undergo small conformational changes after heme binding. Even though the aforementioned studies provided a wealth of information on heme–protein interactions and gave some invaluable insights into computational prediction of key residues involved in these interactions, no algorithm has been developed for specifically detecting heme-binding residues, which would be very useful in predicting and designing novel heme proteins and helping to illuminate heme binding mechanisms. Accordingly, our group [14] proposed the first specialized algorithm, HemeBind, to identify these important residues. This method was developed by combining support vector machines with a group of sequence and structural features, such as evolutionary conservation, solvent accessibility, depth and protrusion. Despite its encouraging performance, the prediction accuracy remains to be further improved. It is thus desirable to find new features that can well characterize heme- binding residues and complement conventional features for predicting these residues.

On the other hand, there has been increasing interest in studying proteins by representing their three-dimensional structures as residue interaction networks and analyzing the topological properties of functionally important residues. Greene et al. [15] and Bagler et al. [16] showed the small-world and scale-free properties of protein residue networks, and further validated that these attributes are independent of the protein structural class. Vendruscolo et al. [17], [18] found that key residues in the process of protein folding generally correlate with residues having larger connectivity values in a residue network. Brinda and Vishveshwara [19] demonstrated that hub residues in protein structures usually play a critical role in protein folding and stability. Additionally, Amitai et al. [20] revealed that active site residues in enzymes tend to have higher closeness values and developed a method that effectively identified these residues by combining closeness and surface accessibility. Del Sol et al. [21], [22] showed that protein complexes can also be represented as small-world networks and used this fact to predict the hot spots in protein–protein interfaces. Recently, Li et al. [23] characterized non-synonymous single nucleotide polymorphisms (nsSNPs) by residue interaction networks and predicted these crucial residues using topological features. In summary, the application of network concepts has significantly enhanced our understanding of protein structure, function and dynamics. However, to the best of our knowledge, there is no study that conducted a systematic characterization of ligand-binding sites from the network perspective and tried to predict ligand-binding residues based on their topological features.

This study aims to explore the possibility of utilizing the topological information extracted from residue interaction networks to identify the binding residues of heme ligands in protein structures. We found that four well-established network-based features, including degree, closeness, betweenness and clustering coefficient, can be used to well characterize heme-binding residues. To predict these critical residues, we developed HemeNet, a support vector machine based algorithm by integrating topological features with various sequence and structural features, which significantly improved the prediction performance. In addition, we demonstrated that these network-based features can effectively depict the heme-binding regions of apo structures as well as those of holo structures. Moreover, incorporation of these features also improved the accuracy for predicting the binding residues of heme proteins in their free state. The proposed method provides an additional way to characterize heme-binding residues and could aid in improving other ligand-binding residue prediction.

## Materials and Methods

### Data collection

Three datasets prepared in [14] were used in the current study. For convenience, the main, alternative and independent test datasets were renamed as Dataset 1, Dataset 2 and Dataset 3, respectively. Dataset 1 is a non-redundant set composed of 141 heme proteins with mutual sequence identity less than 30%. Dataset 2 contains 75 non-redundant heme proteins originally prepared by Fufezan et al. [11], where no two chains share more than 25% sequence identity. Dataset 3 including 62 single-heme and 10 multi-heme proteins was used for independent testing. In addition, Li et al. [13] collected 10 non-redundant holo-apo heme protein pairs with high sequence similarity recently which we called Dataset 4. This dataset gave us a chance to further test our algorithm on the unbound form of heme proteins. More details about these four datasets can be found in related references.

In our previous study, all residues of heme proteins were considered as the potential heme-binding residues. Currently, we have only reserved the residues with a non-zero solvent accessible surface area, considering that few of the totally buried residues in heme proteins are in contact with heme ligands and thus we can directly skip them when predicting heme-binding residues. On the other hand, compared to the rest of the protein, these buried residues usually have higher topological attribute values, such as degree and closeness [20], [24], which may result in a bias for characteristics analysis if they were considered as non-binding residues. Accordingly, as we defined the binding interface for each heme protein, the residues with a zero accessible surface area were filtered out, and the remaining residues were then divided into binding and non-binding groups using the Ligand Protein Contact (LPC) server [25]. Table 1 shows detailed information about the four datasets used in this study.

**Table 1:** Summary of four datasets used in this study.

Dataset	Chains	Binding Residues	Non-binding Residues	Ratio^a^
Dataset 1	141	5035	29234	14.7%
Dataset 2	75	2490	14376	14.8%
Dataset 3	72	2632	14167	15.7%
Dataset 4	10 (10)^b^	252 (217)	2210 (2088)	10.2% (9.4%)

a Ratio = number of binding residues/(number of binding residues+number of non-binding residues).

b The information of apo heme proteins used in Dataset 4.

### Network-based feature extraction

The three-dimensional structure of each heme protein can be converted into a residue interaction network, in which residues are denoted as nodes and the contacts between them are denoted as edges. Here, residue *i* is considered to be in contact with residue *j* if the distance between any heavy atom of residue *i* and that of residue *j* is less than 5 Å. This cutoff approximates the upper limit for attractive London-van der Waals forces [15]. Further, the residue network can be expressed by its adjacency matrix *A* with an element is equal to 1 if residues *i* and *j* are in contact and 0 otherwise. Based on the adjacency matrix, four well known network-based measures, such as degree, closeness, betweenness and clustering coefficient, were used to describe the topological characteristics of each residue in a given protein structure. The detailed description of these features is given below.

#### Degree

Degree is a commonly used measure to reflect the local connectivity of a node. In a residue interaction network, the degree of residue *i* is the number of its direct connections to other residues and can be calculated as:
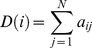
(1)where *a_ij_* is the element of adjacency matrix *A*, and *N* is the total number of nodes in the residue network.

#### Closeness

Closeness is a global centrality metric used to determine how critical a residue is in a residue interaction network. The closeness of residue *i* is defined as the inverse of the average geodesic distance (shortest path) from residue *i* to all other residues in the network. Generally, the residues with shorter geodesic distances to the remaining residues tend to have higher closeness values. The shortest paths between all pairs of residues were identified using the Dijkstra's algorithm [26]. The closeness values can be calculated as:
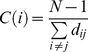
(2)where *d_ij_* is the shortest path from residue *i* to residue *j*, and *N* is the total number of nodes.

#### Betweenness

Betweenness is another important global centrality measure for a residue in our study. The betweenness of residue *i* is defined to be the sum of the fraction of shortest paths between all pairs of residues that pass through residue *i*. Hence the residues that occur more often on the shortest paths between other residues should have a higher betweenness than those that do not. The betweenness values should be normalized by the total number of residue pairs as:
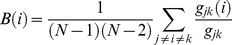
(3)where *g_jk_* is the number of shortest paths between residues *j* and *k*, and *g_jk_(i)* is the number of these shortest paths passing through residue *i*.

#### Clustering coefficient

The clustering coefficient of a residue is a local measure that quantifies how close its neighbors are to being a clique. The clustering coefficient of residue *i* is given by the proportion of connections between the neighboring residues divided by the maximum possible connections within the neighborhood and can be represented as:
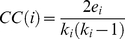
(4)where *e_i_* is the number of edges between the neighbors of residue *i*, and *k_i_* is the number of its neighbors.

#### Standardization

To make the topological features comparable among different proteins, the raw attribute values should be converted into z-scores for each residue as follows:
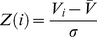
(5)where *V_i_* is the attribute value of residue *i* for a given topological feature, 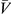 is the average value over all residues in a given protein structure, and σ is the standard deviation.

### Conventional feature extraction

In addition to the network-based attributes, several sequence and structural features widely used in protein functional site prediction were also extracted to depict the residues located in heme-binding interfaces, including sequence profile, solvent accessibility, depth and protrusion. The detailed description of these features is given below.

#### Position specific scoring matrix (PSSM)

PSSM is commonly used to reflect the residue evolutionary conservation in a particular protein of interest. The PSI-BLAST program [27] was used to generate the PSSM of amino acid sequences with parameters j = 3 and e = 0.001. The search was performed against the non-redundant (NR) database from NCBI.

#### Relative accessible surface area (RASA)

Accessible surface area (ASA) is the atomic surface area of a molecule that is accessible to solvent. The DSSP program [28] was used to calculate the ASA value of each residue in the unbound chain. To obtain the RASA of each residue, the ASA value was divided by the maximum ASA of its residue type in a tri-peptide state [29].

#### Depth index (DPX) and protrusion index (CX)

DPX and CX are important metrics used to describe the geometric shape of a protein, which measure the local concavity and convexity of the protein surface respectively. In our study, the PSAIA software [30] with default parameters was utilized to generate the DPX- and CX-related features of each residue in the unbound chain, including the average and standard deviation of all atom values, the average and standard deviation of all side-chain atom values, and the minimal and maximal atom values.

### Prediction model construction

In this work, support vector machine (SVM) based classifiers are proposed to identify key residues involved in heme-binding interfaces. Based on the nature of the features aforementioned, they can be divided into three subsets: (i) network-based features (including degree, closeness, betweenness and clustering coefficient); (ii) geometry-based features (including solvent accessibility, depth and protrusion); (iii) conservation-based features (including sequence profile). These three feature subsets were then used separately or combined to construct the SVM predictors by integrating with a structural window composed of the target residue and its 14 spatially nearest residues. In order to benchmark the new algorithm, we chose the predictor based on the conservation- and geometry-based feature subsets as the baseline model, which was the default feature combination of the structure-based predictor in our previous study. To implement our algorithm, the LIBSVM package [31] was used to build the predictors and the radial basis function was chosen as the kernel. It is worth mentioning that with the exception of the RASA feature, the remaining features should be scaled to the range [0, 1] using the standard logistic function. The values of C and γ were 2 and 0.03125 for all predictors respectively.

### Performance evaluation

To validate the effectiveness of our method, we first tested our algorithm on Dataset 1 and Dataset 2 by a 5-fold cross-validation. The protein chains were randomly divided into five subsets, four of which were used for training and the remaining one for testing. In order to overcome the imbalance issue of positive and negative samples, we used all heme-binding residues and an equal number of randomly extracted non-binding residues for training the predictors in each validation. Furthermore, Dataset 3 and Dataset 4 were used as the independent test sets to check our prediction models. Here, recall, precision, accuracy, F1-score and Matthews correlation coefficient (MCC) were adopted for model evaluation. The definitions of these measures are given in our previous study. In addition, the receiver operating characteristic (ROC) curve, in which one plots false positive rate on the *x*-axis against true positive rate on the *y*-axis in terms of different prediction thresholds, was used to evaluate the overall performance. The area under the ROC curve (AUC) was also calculated to assess the robustness of our method. Generally, an AUC value closer to 1 indicates a better prediction performance.

### Statistical inference

The student's *t*-test was used to check whether there is a significant difference for a given property between heme-binding residues and other residues on the protein surface. To evaluate the potential discriminatory power of this property, we calculated its F-score as defined below [32]:
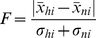
(6)Where 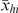 and 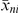 are the averages over the heme-binding and non-binding groups, and 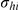 and 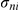 are the corresponding standard deviations. The F-score reflects the separation of means for two populations according to their variances. Additionally, the Wilcoxon signed-rank test was applied to assessing the statistically significant difference between paired predictors in this work.

## Results and Discussion

### Network-based features of heme-binding residues

The main idea of our algorithm is based on the incorporation of novel topological properties extracted from residue interaction networks to improve the prediction of heme-binding residues. We first sought to examine whether the network-based features have the potential predictive capability in distinguishing the heme-binding residues from the rest of non-buried residues. Moreover, we compared the network features with other conventional features. To address this problem, the residues in Dataset 1 were divided into binding and non-binding groups, and the *t*-test combined with the F-score estimation were used to assess the discriminatory power of each individual feature.

As shown in Table 2, the mean values of degree, closeness and betweenness measures for binding residues were significantly higher than those of non-binding residues, suggesting that the residues with greater connectivity and/or centrality values in residue interaction networks are more likely to be involved in the binding of heme ligands. This is in line with the topological knowledge about ligand-binding sites reported by other groups. For example, for the degree measure, Illingworth et al. [33] observed that the residues within ligand-binding regions have about 25% more contact neighbors than surface residues in general, and they gave a possible rationale that there will be the less loss of conformational entropy on ligand binding. On the other hand, Amitai et al. [20] demonstrated that ligand-binding residues have typically high closeness values for several well-studied protein structures. In addition, Del Sol et al. [34] revealed that in the 33 protein families binding hetero-atoms, 64% of the centrally conserved residues related to the residues in hetero-atoms binding sites. The main reason for ligand-binding residues generally having high centrality values might be that these residues (including heme-binding residues) are usually located in the largest pockets or clefts which are closer to the protein center of mass than the non-binding surface. So they could fulfill important roles in integrating and propagating information to the remaining residues of the protein. Interestingly, in this study we found that compared with non-binding residues, the mean clustering coefficient of heme-binding residues was obviously lower. This is probably owing to the fact that the neighborhoods of residues in the binding pockets are less packed relative to the rest of the protein, allowing a certain degree of flexibility for heme-binding sites.

**Table 2:** Comparison of the potential predictive capability of different features.

Feature	F-score	Mean ± SD (Binding)	Mean ± SD (Non-binding)	P-value
Degree	0.23	0.27±0.92	−0.17±0.96	1.62×10^−199^
Closeness	0.39	0.59±1.01	−0.18±0.95	0
Betweenness	0.38	0.67±1.30	−0.16±0.88	0
Clustering Coefficient	0.30	−0.41±0.81	0.14±1.03	0
Sequence Conservation^a^	0.25	4.69±3.40	3.17±2.65	5.45×10^−188^
Solvent Accessibility	0.19	0.26±0.21	0.35±0.27	1.82×10^−159^
Depth^b^	0.06	0.69±0.57	0.62±0.58	1.92×10^−16^
Protrusion^c^	0.20	0.53±0.53	0.77±0.68	2.83×10^−172^

a The diagonal element of PSSM at each residue position was used to measure the conservation of each residue.

b The average of all atom DPXs was used to measure the depth of each residue.

c The average of all atom CXs was used to measure the protrusion of each residue.

**Figure 1:**
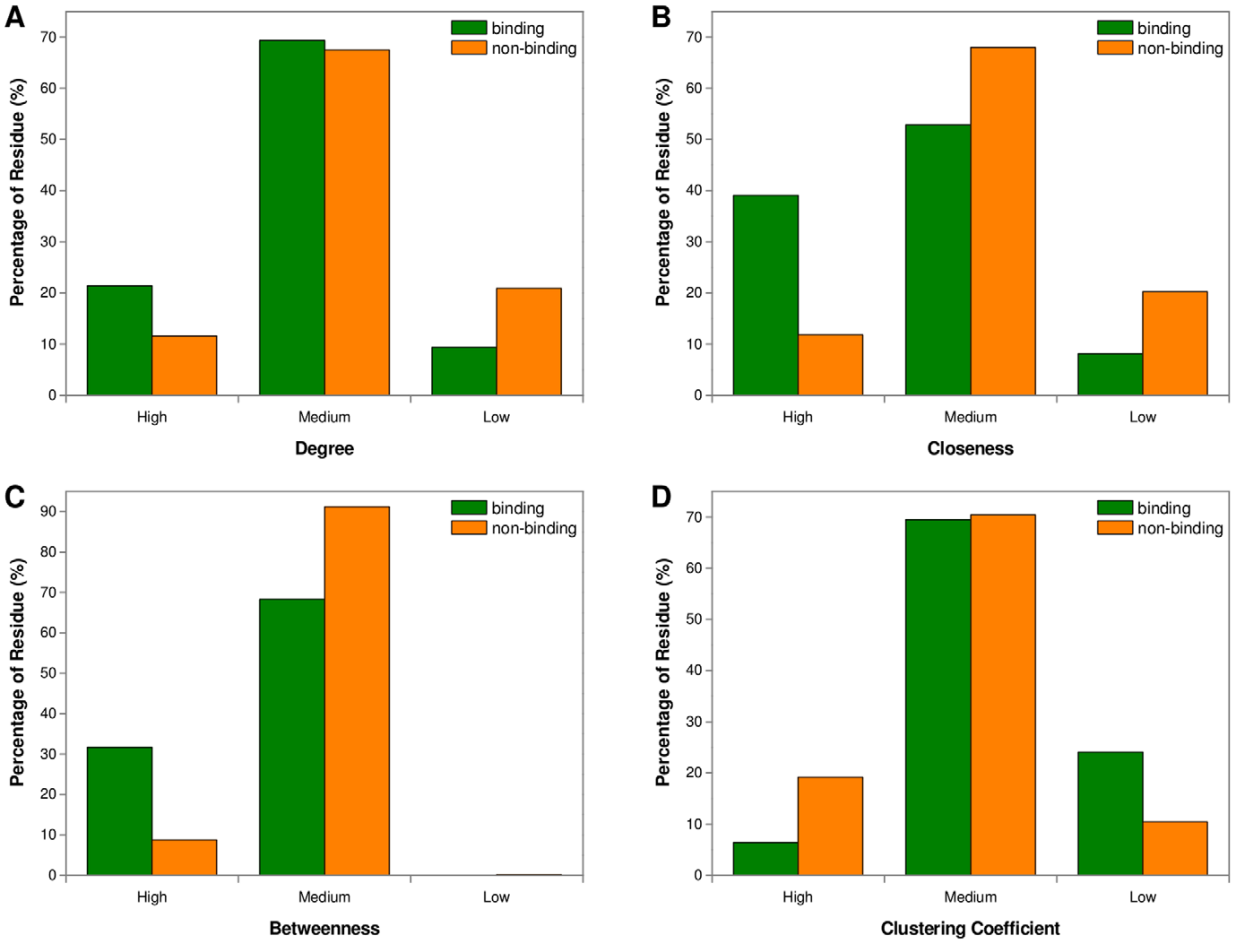
Distribution comparison of network-based features of heme-binding and non-binding residues. (a) Degree; (b) Closeness; (c) Betweenness; (d) Clustering Coefficient. For each topological feature, the z-scores of binding and non-binding residues in Dataset 1 are divided into high, medium and low score sections, and then the fraction occupied by each section was calculated.

To clearly demonstrate the distributions of network-based features, we classified all residues into three sections based on the z-scores of each topological property as following: high score (z-score≥1), medium score (−1≤z-score<1), and low score (z-score<−1). From Figure 1, we see that heme-binding residues appeared more frequently than non-binding residues in the high score section of degree, closeness and betweenness measures. Instead, for the clustering coefficient measure, the binding residues had a relatively higher proportion in the low score section. These results further confirmed, to some extent, the network-based features can be used to quantitatively depict the difference between heme-binding regions and the rest of the protein surface.

We also compared the mean values of other sequence and structural features in Table 2. The differences of these conventional features for binding and non-binding residues are largely consistent with our previous study, with the exception of the depth feature. Here, the average depth of binding residues was slightly higher compared to that of non-binding residues. This is mainly due to the fact that the totally buried residues with greater depth values were not considered as non-binding residues in this work. On the other hand, considering the F-scores of different attributes, we found that the F-score of the degree feature was just slightly lower than that of evolutionary conservation, which is widely considered to be the most important feature for protein functional residue prediction in existing studies. Furthermore, the F-scores of the other three network-based features were clearly greater than those of the conventional features. This result suggested that the new topological features should have the potential capability for identifying heme-binding residues.

**Figure 2:**
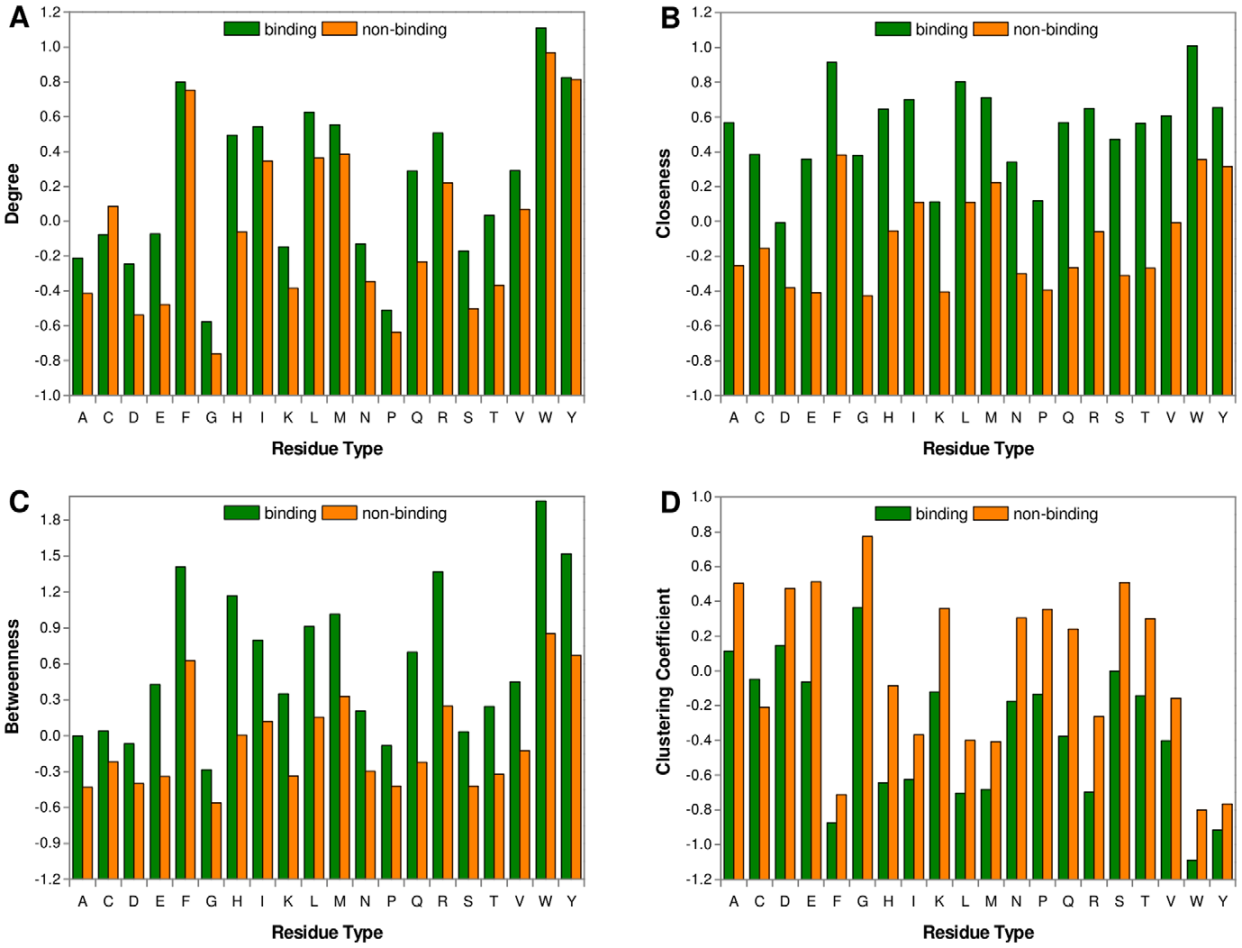
Comparison of network-based features of heme-binding and non-binding residues based on residue type. (a) Degree; (b) Closeness; (c) Betweenness; (d) Clustering Coefficient. For each topological feature, we compared the means of the z-scores of binding and non-binding residues in Dataset 1 for different residue types.

Focusing on the network-based features, we compared the average values of the binding and non-binding groups for each residue type. The results are presented in Figure 2 and Table S1. It can be seen from Figure 2A that except for cysteine, the binding residues had greater average degrees for the remaining residue types. Figure 2B–C indicated that for all residue types, the average closeness and betweenness values of heme-binding groups were obviously higher than those of non-binding groups. Figure 2D showed that the average clustering coefficients of all residue types except for cysteine were relatively lower in heme-binding regions. These results demonstrated that the differences between binding and non-binding residues in these topological measures do not have a preference for specific residue types. Taken together, the analysis of potential predictive power for topological features implied that they could be combined with existing sequence and structural features to improve the prediction of heme-binding residues.

### Performance of 5-fold cross-validation

**Figure 3:**
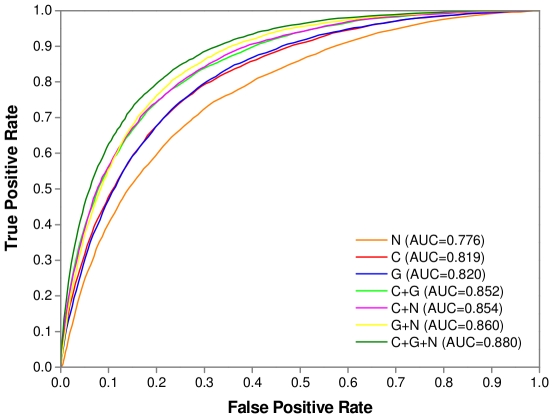
The ROC curves of different predictors tested on Dataset 1. Seven SVM-based predictors were built in terms of different feature combination (N/G/C = Network/Geometry/Conservation) and evaluated by a 5-fold cross-validation on Dataset 1.

To systematically evaluate the usefulness of different feature subsets, seven SVM predictors were built based on different feature combinations. The prediction results tested on Dataset 1 by a 5-fold cross-validation are summarized in Table 3. It can be seen that when these feature subsets were used individually, the conservation- and geometry-based features achieved a similar performance, with a F1-score of about 48% and MCC of about 0.38. However, the network-based feature subset did not perform as well as the other two subsets. Even so, we obtained a promising result with a F1-score of 43.43% and MCC of 0.323, which indicates that the topological features could be helpful in distinguishing heme-binding residues from non-binding residues. Further, as different subsets were combined for prediction, the three predictors based on the combination of two subsets all demonstrated a remarkably better performance compared to the predictors based on individual subset. Interestingly, although we observed from Table 3 that the optimal performances of these three predictors were similar, the ROC curves in Figure 3 showed that the predictor based on network- and geometry-based features, a purely structure-based prediction model, slightly outperformed the other two predictors in terms of overall performance. This result indicated that the topological features in conjunction with other structural information could effectively recognize the potential binding sites in heme proteins without enough homologs and result in considerable savings in computational time. Finally, if all the three subsets were combined, the predictor yielded the best result with a F1-score of 56.76% and MCC of 0.489. We called this predictor HemeNet. In particular, HemeNet achieved a significantly better performance than the baseline model considering conventional features alone (Wilcoxon signed-rank test, *p*-value = 1.51×10^−181^). The recall, precision and F1-score increased by about 4% respectively, and the MCC value raised by about 5%. Similar improvement was observed as Dataset 2 was tested and the results are given in Table S2 and Figure S1. In the baseline model, the geometry-based features have reflected the local structural characteristics of a given residue. In addition to the local consideration, HemeNet took advantage of the topological features, especially closeness and betweenness, which measured the importance of this residue in the global structure, giving rise to the improved performance. In Figure 3, we clearly showed the ROC curves and AUCs of different predictors. This highlighted that the network-based features are distinct from various sequence and structure features and can thus complement them in the prediction of heme-binding residues.

**Table 3:** Performance of different predictors tested on Dataset 1.

Feature Set	Recall (%)	Precision (%)	Accuracy (%)	F1-score (%)	MCC
Network	55.71	35.61	78.69	43.43	0.323
Geometry	55.87	41.77	82.08	47.78	0.378
Conservation	56.48	41.60	81.90	47.87	0.379
Geometry+Network	59.37	47.31	84.32	52.62	0.438
Conservation+Geometry	58.72	47.98	84.52	52.72	0.440
Conservation+Network	58.84	48.11	84.60	52.92	0.442
Conservation+Geometry+Network	62.70	52.05	85.99	56.76	0.489

It should be pointed out that as 5-fold cross-validation was conducted on Dataset 1, the structural homologs in this dataset were not removed. To further test the robustness of our method, we collected a structurally non-redundant dataset by following Li et al.'s method [13]. In Dataset 1, we identified a total of 66 protein chains with SCOP annotations and belonging to 26 distinct structural folds. We then collected 26 chains by randomly selecting one chain from each fold and conducted cross-validation on these chains. As expected, there was a dramatic decrease in the performances of both the baseline model and HemeNet according to Table S3. This result indicated that utilizing structural similarity is critical for achieving accurate heme-binding residue prediction. Compared with the baseline model, however, HemeNet still significantly raised the F1-score and MCC values by about 4% respectively, suggesting that incorporation of topological features can help to improve the prediction performance in this challenging case with much less structural redundancy.

### Independent testing on single- and multi-heme proteins

**Figure 4:**
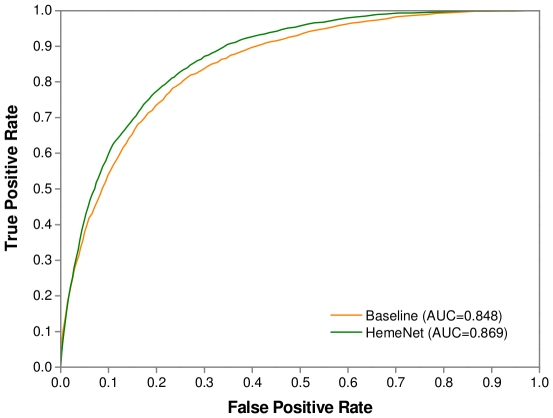
The ROC curves of baseline model and HemeNet tested on Dataset 3. The baseline model was the predictor considering only conventional features, while HemeNet incorporated network-based features into the baseline model.

As is well known, heme proteins can interact with either a single heme molecule or multiple heme molecules. Accordingly, it was interesting to examine whether the network-based features can be used to improve the accuracy for predicting binding residues in both types of heme proteins. Herein we used Dataset 2 as a training set to train the baseline model and HemeNet, and evaluated their performances based on Dataset 3. As demonstrated in Table 4, by incorporating the topological features, the F1-score and MCC were increased from 46.63% to 51.08% and 0.388 to 0.439 for single-heme proteins, respectively. On the other hand, for multi-heme proteins, the performance of the baseline model was much better than that obtained on single-heme proteins, which was in agreement with the observation in our previous study. However, compared to the baseline model, HemeNet modestly improved the prediction accuracy, yielding an approximate 3% increase in the F1-score and MCC values, respectively. These results indicated that the incorporation of network-based features is beneficial for identifying the binding residues of both single- and multi-heme proteins. Additionally, for Dataset 3 the HemeNet algorithm also significantly outperformed the baseline model (Wilcoxon signed-rank test, *p*-value = 3.82×10^−126^), with a F1-score of 56.42% and MCC of 0.477 which was just marginally lower compared to the performance of 5-fold cross-validation on Dataset 1. The ROC curves in Figure 4 further confirmed the advantage of HemeNet over the baseline model in terms of overall performance on this dataset.

**Table 4:** Performance of baseline model and HemeNet tested on Dataset 3.

Subset	Model	Recall (%)	Precision (%)	Accuracy (%)	F1-score (%)	MCC
Single-heme	Baseline	53.29	41.46	85.43	46.63	0.388
	HemeNet	59.44	44.77	86.40	51.08	0.439
Multi-heme	Baseline	66.77	61.30	74.91	63.92	0.448
	HemeNet	70.46	62.74	76.24	66.38	0.483
All	Baseline	58.28	48.06	83.59	52.68	0.432
	HemeNet	63.53	50.74	84.62	56.42	0.477

### Network-based feature comparison between holo and apo heme proteins

Recently, Li et al. [13] collected 10 holo-apo heme protein pairs and checked the conformational differences between the holo and apo protein structures. They demonstrated that 9 out of 10 proteins had very small global conformational changes after heme binding with RMSDs (root mean square deviations) of 1.03 Å or less. Intuitively, we would expect that the topological structure of residue interaction networks of heme proteins should also undergo small changes upon heme–protein complex formation, and thus the network-based features could well characterize the binding residues of apo structures as well as those of holo structures. To validate this hypothesis, we calculated the average values of each topological feature for heme-binding and non-binding residues and compared the average values of each holo-apo protein pair. A detailed comparison is given in Table 5. Compared with non-binding residues in the holo structures, we found that the heme-binding residues of each individual protein generally had higher averages of degree, closeness and betweenness, but a lower mean of clustering coefficient. This was consistent with the results obtained by analyzing the topological features on the whole Dataset 1. More importantly, as expected, similar phenomena were also observed on the apo structures, suggesting that the network-based features indeed pre-exist in the unbound form of heme proteins.

**Table 5:** Comparison of network-based features of holo-apo heme protein pairs.

Holo Chain	Residue Number	Degree	Closeness	Betweenness	Clustering Coefficient	Apo Chain	Residue Number	Degree	Closeness	Betweenness	Clustering Coefficient
1KBI:A	31^a^	0.30	0.47	0.54	−0.62	1SZF:B	11	0.01	0.23	−0.04	−0.11
	413^b^	−0.17	−0.13	−0.07	0.13		324	−0.16	−0.14	−0.06	0.11
1N45:A	25	0.41	0.80	0.89	−0.26	1S8C:D	25	0.31	0.63	0.80	−0.24
	166	−0.21	−0.24	−0.22	0.14		166	−0.19	−0.22	−0.21	0.13
1N5U:A	24	0.26	0.95	1.03	−0.50	3CX9:A	24	0.19	0.74	0.53	−0.38
	532	−0.07	−0.05	−0.04	0.06		530	−0.06	−0.04	−0.01	0.04
2ITF:A	19	−0.20	−0.46	0.13	0.03	2ITE:B	19	−0.25	−0.48	0.14	0.12
	96	−0.03	0.01	−0.14	0.04		96	−0.03	0.01	−0.14	0.02
2NWB:A	29	0.41	1.07	0.89	−0.43	1ZEE:B	26	0.22	1.04	0.84	−0.32
	311	−0.18	−0.16	−0.13	0.12		300	−0.14	−0.13	−0.10	0.10
2OFR:X	28	0.49	0.87	0.99	−0.59	2OFM:X	28	0.55	0.93	1.01	−0.57
	143	−0.19	−0.26	−0.25	0.16		143	−0.20	−0.26	−0.25	0.16
2R7A:A	22	−0.09	1.04	1.23	−0.35	2RG7:D	22	−0.25	0.69	0.88	−0.14
	202	−0.14	−0.19	−0.17	0.13		206	−0.10	−0.11	−0.11	0.09
2ZDO:A	25	0.42	0.89	1.06	−0.72	1XBW:D	23	0.37	0.79	0.93	−0.60
	79	−0.22	−0.32	−0.38	0.27		71	−0.22	−0.29	−0.35	0.24
3CQV:A	24	−0.06	0.37	0.68	−0.49	2V7C:A	16	0.16	0.29	0.63	−0.32
	150	−0.12	−0.13	−0.16	0.16		133	−0.11	−0.11	−0.14	0.09
3EMM:A	25	0.23	0.01	0.48	−0.32	2A13:A	23	0.04	−0.10	0.49	−0.26
	118	−0.15	−0.07	−0.17	0.13		119	−0.10	−0.05	−0.15	0.10

a The number of heme-binding residues in each heme protein and the mean topological parameters of these residues are given in the upper row.

b The number of non-binding residues in each heme protein and the mean topological parameters of these residues are given in the lower row.

**Figure 5:**
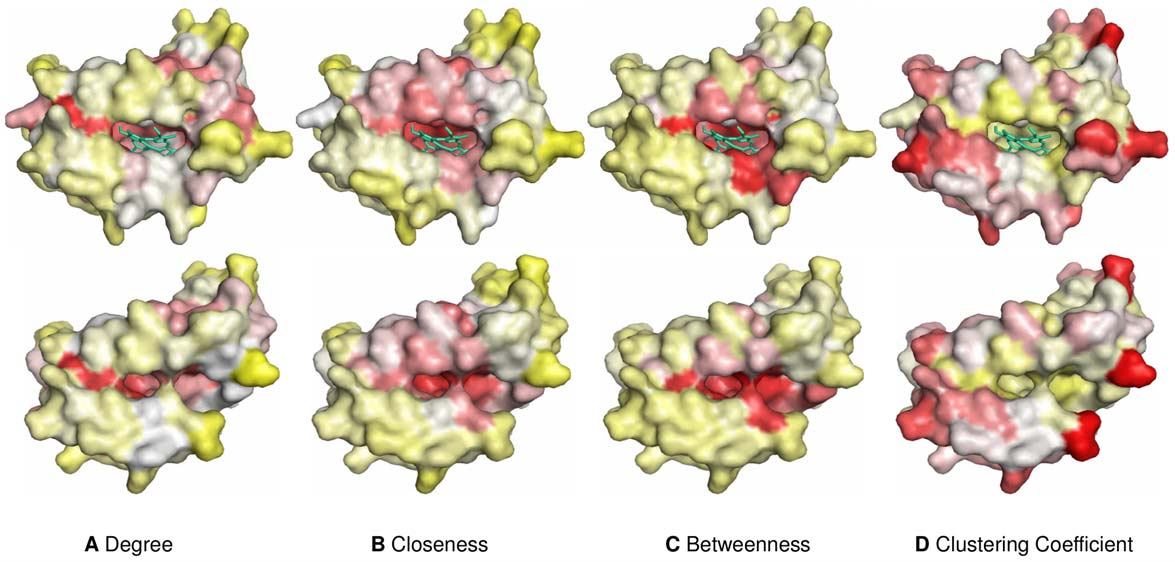
Visualization of the surface of holo-apo protein pair (2ZDO:A-1XBW:D). (a) Degree; (b) Closeness; (c) Betweenness; (d) Clustering Coefficient. The color of the surface was changed according to the z-scores of each network-based feature (red/white/yellow = high/medium/low). The heme molecule was shown as a cyan stick. The holo structures (2ZDO:A) were presented in the upper row, and the apo structures (1XBW:D) were presented in the lower row. This figure was produced by PyMOL (www.pymol.org).

In most cases, the average degree, closeness and betweenness values of binding residues were obviously higher in the holo structures compared to those in the apo structures, whereas the average clustering coefficient value was relatively lower. The increases in the connectivity and centrality values further confirmed the important role of binding residues in forming heme–protein complexes. Conversely, for non-binding residues, with the exception of the clustering coefficient, the means of the remaining topological measures were slightly smaller in the holo structures. As a result, the discrepancy between the average topological measures of heme-binding group and those of non-binding group became more apparent after heme binding. For example, the protein pair 2ZDO:A-1XBW:D with a RMSD of 0.59 Å clearly showed this change. In the apo structure (1XBW:D), the differences between binding group and non-binding group were 0.59, 1.08, 1.28 and 0.84 (absolute value) for degree, closeness, betweenness and clustering coefficient measures, respectively. Upon complex formation, the corresponding values increased to 0.64, 1.21, 1.44 and 0.99 (absolute value) in the holo structure (2ZDO:A), which reflected the structural change from the topological perspective of residue interaction network. Figure 5 demonstrated the three-dimensional structures of this holo-apo protein pair in terms of network-based attribute values. We can see that the four topological features of the heme-binding pocket unambiguously differed from those of the remaining protein surface for both holo and apo structures. Overall, the aforementioned analysis implied that the topological information in the residue interaction network should be helpful for the identification of binding residues in the heme-free state.

### Independent testing on holo and apo heme proteins

**Figure 6:**
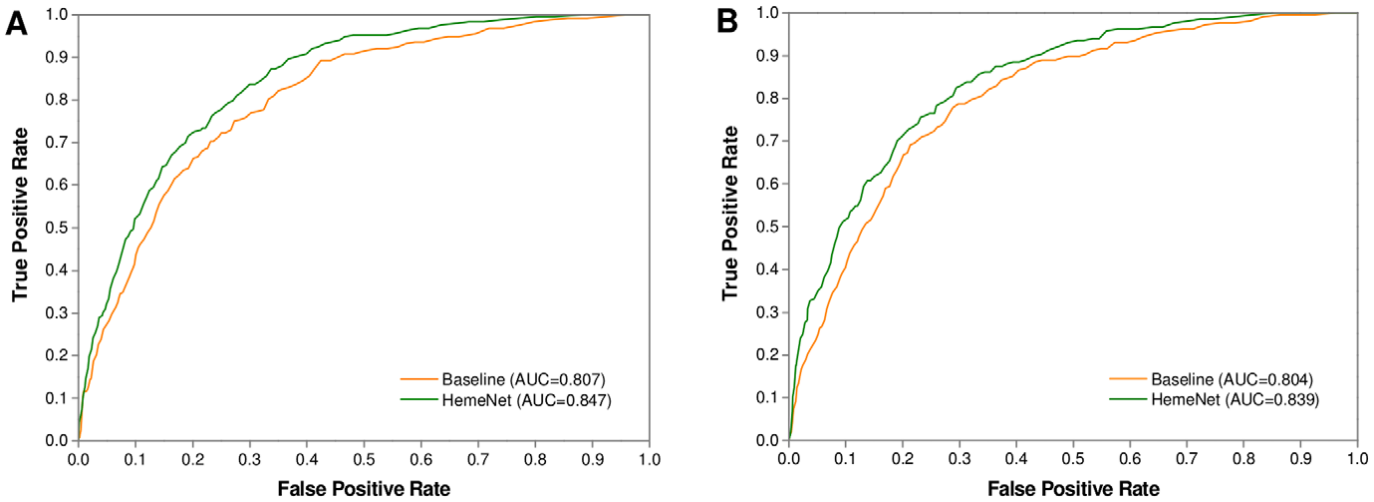
The ROC curves of baseline model and HemeNet tested on Dataset 4. (a) Holo structures; (b) Apo structures.

In this section, we checked the performance of HemeNet on Dataset 4 compared with that of the baseline model. To train these two predictors, the non-homologous chains in Dataset 1 that share less than 30% sequence identity with any chain in Dataset 4 were retrieved. Owing to the fact that the number of chains in this dataset is relatively few, besides the overall measures generally used in this paper, we applied the average measures to evaluating the performance. The overall measures denote that the measures were calculated based on the total predictions of all proteins, while the average measures mean that the measures were obtained by averaging the performance of each protein. As shown in Table 6, when we just extracted sequence and structural features for prediction, the overall F1-score and MCC values for the apo and holo structures were 35.51% and 0.280, and 38.30% and 0.305, respectively. However, if the network-based features were incorporated into the baseline model, the corresponding measures significantly increased to 41.58% and 0.351 for the apo structures (Wilcoxon signed-rank test, *p*-value = 2.08×10^−17^), and to 43.92% and 0.373 for the holo structures (Wilcoxon signed-rank test, *p*-value = 5.88×10^−16^). Furthermore, when the average measures were considered as evaluation metrics, the prediction performance was better and similar improvements were also observed. More concretely, we can see that except for 1SZF:B and 2ITE:B, the F1-score and MCC values of the remaining apo chains and all holo chains were improved to a certain degree in Table S4, S5. In addition, the ROC curves and AUCs of these two predictors are shown in Figure 6. According to the results given in Table 6 and Figure 6, we can conclude that the baseline and HemeNet models are both insensitive to the conformational changes triggered by heme binding, and that the use of topological features indeed effectively improved the prediction accuracy for both holo and apo structures.

**Table 6:** Performance of baseline model and HemeNet tested on Dataset 4.

	Baseline		HemeNet	
	Holo	Apo	Holo	Apo
Recall (%)	50.00 (48.58)^a^	43.78 (43.21)	58.73 (57.77)	53.46 (51.95)
Precision (%)	31.03 (39.41)	29.87 (38.56)	35.07 (42.01)	34.02 (40.64)
Accuracy (%)	83.51 (83.93)	85.03 (85.00)	84.65 (84.51)	85.86 (85.25)
F1-score (%)	38.30 (40.19)	35.51 (36.90)	43.92 (45.93)	41.58 (42.95)
MCC	0.305 (0.336)	0.280 (0.311)	0.373 (0.395)	0.351 (0.366)

a The average measures of holo-apo protein pairs in Dataset 4.

### Comparison with ConCavity

**Figure 7:**
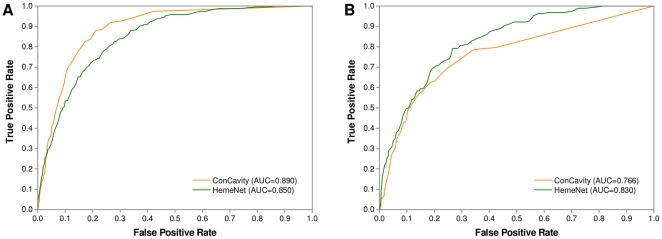
The ROC curves of ConCavity and HemeNet tested on holo-apo protein pairs. (a) Holo structures; (b) Apo structures.

To further show the effectiveness of the proposed algorithm, we compared HemeNet with ConCavity [35] based on Dataset 4, which was one of the state-of-art algorithms in general ligand-binding residue prediction by incorporating residue evolutionary conservation into pocket detection. In Dataset 4, one holo-apo structure pair and three apo structures have no prediction results in the web server of ConCavity. However, for these three apo structures, the results of their identical chain in the same complex can be downloaded. Hence, to make a fair comparison, we replaced the original chains with their identical chains to test HemeNet. From Table 7 and Figure 7, we can see that ConCavity remarkably outperformed HemeNet for the holo structures. However, when the apo structures were tested, there was a dramatic decrease in its prediction performance, indicating that the ConCavity algorithm is sensitive to conformational changes in heme-binding regions. Compared with ConCavity, HemeNet achieved a better performance on the apo structures. The main reason might be that network-based features not only uniquely reflect the role of binding residues in the global structure, but also tolerate a certain degree of conformational change similar to conventional features. As expected, we found that the performance discrepancy of HemeNet between the holo and apo structures was much smaller than that of ConCavity. Since the final aim of binding residue prediction should be finding the potential binding residues in the apo structures, the HemeNet algorithm has its advantage by considering the topological features derived from residue interaction networks. Accordingly, these features could provide some complementary information for existing ligand-binging residue prediction algorithms.

**Table 7:** Performance comparison of ConCavity and HemeNet.

	ConCavity		HemeNet	
	Holo	Apo	Holo	Apo
Recall (%)	58.80	52.60	59.23	54.17
Precision (%)	42.02	29.36	34.24	30.77
Accuracy (%)	87.86	84.70	84.66	85.25
F1-score (%)	49.02	37.69	43.40	39.25
MCC	0.431	0.314	0.370	0.332

### Conclusions

We have applied topological features to heme-binding residue prediction by representing protein structures as residue interaction networks. It was found that network-based features can be used to effectively characterize heme-binding residues in the networks. By combining these topological features with various sequence and structural features, we significantly improved the performance of heme-binding residue prediction. In addition, due to the small conformational changes of heme proteins after ligand binding, the topological features can also be used to quantitatively depict the binding regions in apo structures, resulting in the prediction of binding residues in the heme-free state achieving a reasonable enhancement. In conclusion, the topological features extracted from residue interaction networks suggest a new way to characterize heme-binding residues and could provide new insights into general ligand-binding site prediction.

## Supporting Information

Figure S1 The ROC curves of baseline model and HemeNet tested on Dataset 2.
(PDF)

Table S1 Comparison of network-based features based on different residue type.
(PDF)

Table S2 Comparison of the prediction performance on Dataset 2.
(PDF)

Table S3 Comparison of the prediction performance on 26 heme proteins.
(PDF)

Table S4 Comparison of the prediction performance for individual holo structure.
(PDF)

Table S5 Comparison of the prediction performance for individual apo structure.
(PDF)
