# Supplementary figures and images for: Computational Prediction of Heme-Binding Residues by Exploiting Residue Interaction Network
Source: PLoS One. 2011 Oct 3;6(10):e25560. doi: 10.1371/journal.pone.0025560 (PMC3184988; doi:10.1371/journal.pone.0025560)

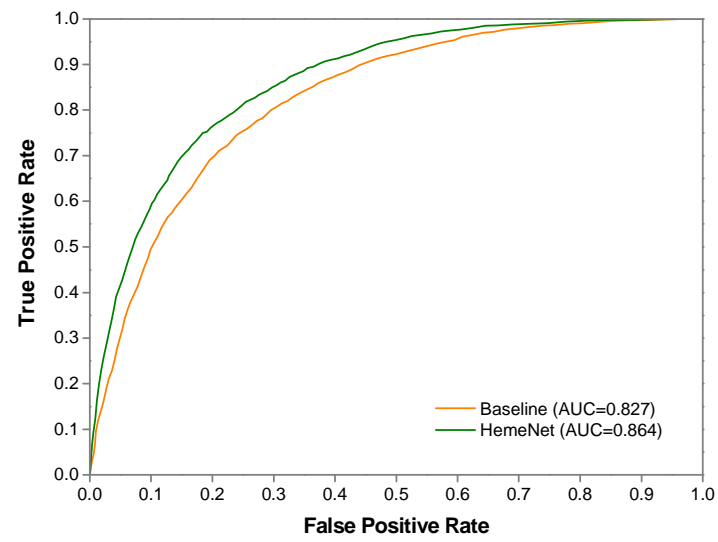

**Figure S1** The ROC curves of baseline model and HemeNet tested on Dataset 2.

Supplement: Figure S1 — The ROC curves of baseline model and HemeNet tested on Dataset 2. (PDF) [file pone.0025560.s001.pdf]
