# Supplementary material for: Computational Prediction of Heme-Binding Residues by Exploiting Residue Interaction Network
Source: PLoS One. 2011 Oct 3;6(10):e25560. doi: 10.1371/journal.pone.0025560 (PMC3184988; doi:10.1371/journal.pone.0025560)
Supplement: Table S1 — Comparison of network-based features based on different residue type. (PDF) [file pone.0025560.s002.pdf]

Table S1 Comparison of network-based features based on different residue type

Residue Type	Degree		Closeness		Betweenness		Clustering Coefficient	
	Binding[a]	Non-binding[b]	Binding	Non-binding	Binding	Non-binding	Binding	Non-binding
A	-0.21 ± 0.69	-0.41 ± 0.75	0.57 ± 0.99	-0.25 ± 0.90	-0.01 ± 0.81	-0.43 ± 0.47	0.11 ± 0.79	0.50 ± 0.92
C	-0.08 ± 0.72	0.09 ± 0.69	0.39 ± 0.91	-0.15 ± 0.96	0.04 ± 0.68	-0.22 ± 0.58	-0.05 ± 0.72	-0.21 ± 0.63
D	-0.25 ± 0.84	-0.54 ± 0.82	-0.01 ± 1.05	-0.38 ± 0.88	-0.07 ± 0.94	-0.40 ± 0.64	0.15 ± 0.96	0.47 ± 0.99
E	-0.07 ± 0.97	-0.48 ± 0.84	0.36 ± 1.15	-0.41 ± 0.90	0.43 ± 1.19	-0.34 ± 0.81	-0.06 ± 1.02	0.51 ± 1.08
F	0.80 ± 0.76	0.75 ± 0.88	0.92 ± 0.90	0.38 ± 0.90	1.41 ± 1.36	0.63 ± 1.20	-0.88 ± 0.58	-0.71 ± 0.72
G	-0.58 ± 0.65	-0.76 ± 0.71	0.38 ± 1.05	-0.43 ± 0.95	-0.28 ± 0.58	-0.56 ± 0.41	0.36 ± 0.69	0.77 ± 0.90
H	0.49 ± 0.86	-0.06 ± 0.91	0.65 ± 0.96	-0.05 ± 0.96	1.17 ± 1.38	0.01 ± 0.98	-0.64 ± 0.69	-0.09 ± 0.91
I	0.54 ± 0.79	0.35 ± 0.80	0.70 ± 0.98	0.11 ± 0.89	0.80 ± 1.07	0.12 ± 0.90	-0.63 ± 0.67	-0.37 ± 0.76
K	-0.15 ± 0.94	-0.38 ± 0.86	0.11 ± 0.94	-0.41 ± 0.85	0.35 ± 1.33	-0.34 ± 0.75	-0.12 ± 0.94	0.36 ± 1.02
L	0.63 ± 0.75	0.36 ± 0.84	0.80 ± 0.96	0.11 ± 0.93	0.91 ± 1.17	0.16 ± 0.89	-0.70 ± 0.55	-0.40 ± 0.80
M	0.55 ± 0.79	0.38 ± 0.92	0.71 ± 1.03	0.22 ± 0.96	1.02 ± 1.30	0.33 ± 1.07	-0.68 ± 0.63	-0.41 ± 0.84
N	-0.13 ± 0.87	-0.35 ± 0.91	0.34 ± 1.06	-0.30 ± 0.94	0.21 ± 0.98	-0.30 ± 0.70	-0.18 ± 0.78	0.30 ± 1.03
P	-0.51 ± 0.71	-0.64 ± 0.77	0.12 ± 1.09	-0.39 ± 0.89	-0.08 ± 0.68	-0.42 ± 0.55	-0.13 ± 0.77	0.35 ± 0.93
Q	0.29 ± 0.86	-0.23 ± 0.90	0.57 ± 1.06	-0.27 ± 0.91	0.70 ± 1.32	-0.22 ± 0.80	-0.38 ± 0.73	0.24 ± 1.01
R	0.51 ± 1.05	0.22 ± 1.01	0.65 ± 0.96	-0.06 ± 0.95	1.37 ± 1.75	0.25 ± 1.29	-0.70 ± 0.86	-0.26 ± 0.96
S	-0.17 ± 0.73	-0.50 ± 0.82	0.47 ± 1.02	-0.31 ± 0.95	0.03 ± 0.75	-0.42 ± 0.60	-0.01 ± 0.82	0.51 ± 0.99
T	0.04 ± 0.79	-0.37 ± 0.85	0.56 ± 1.03	-0.27 ± 0.95	0.24 ± 0.81	-0.32 ± 0.72	-0.14 ± 0.81	0.30 ± 0.94
V	0.29 ± 0.77	0.07 ± 0.80	0.61 ± 0.97	-0.01 ± 0.91	0.45 ± 0.94	-0.13 ± 0.69	-0.40 ± 0.62	-0.16 ± 0.79
W	1.11 ± 0.89	0.97 ± 1.07	1.01 ± 0.96	0.36 ± 0.99	1.96 ± 1.73	0.85 ± 1.40	-1.09 ± 0.54	-0.80 ± 0.88
Y	0.83 ± 0.88	0.82 ± 0.96	0.65 ± 1.00	0.32 ± 0.95	1.52 ± 1.60	0.67 ± 1.27	-0.92 ± 0.62	-0.77 ± 0.73

[a] Mean and SD of heme-binding residues for each residue type.

[b] Mean and SD of non-binding residues for each residue type.
