# Supplementary material for: Computational Prediction of Heme-Binding Residues by Exploiting Residue Interaction Network
Source: PLoS One. 2011 Oct 3;6(10):e25560. doi: 10.1371/journal.pone.0025560 (PMC3184988; doi:10.1371/journal.pone.0025560)
Supplement: Table S2 — Comparison of the prediction performance on Dataset 2. (PDF) [file pone.0025560.s003.pdf]

Table S2 Comparison of the prediction performance on Dataset 2

Model*	Recall (%)	Precision (%)	Accuracy (%)	F1-score (%)	MCC
Baseline	56.61	45.17	82.71	49.47	0.403
HemeNet	61.55	50.05	84.94	54.64	0.465

* Wilcoxon signed-rank test, *p*-value=6.22×10^{-112}
