# Supplementary material for: Computational Prediction of Heme-Binding Residues by Exploiting Residue Interaction Network
Source: PLoS One. 2011 Oct 3;6(10):e25560. doi: 10.1371/journal.pone.0025560 (PMC3184988; doi:10.1371/journal.pone.0025560)
Supplement: Table S3 — Comparison of the prediction performance on 26 heme proteins. (PDF) [file pone.0025560.s004.pdf]

Table S3 Comparison of the prediction performance on 26 heme proteins

| Model* | Recall (%) | Precision (%) | Accuracy (%) | F1-score (%) | MCC |
|---|---|---|---|---|---|
| Baseline | 41.11 | 26.51 | 81.30 | 31.94 | 0.226 |
| HemeNet | 45.16 | 30.08 | 82.58 | 35.75 | 0.272 |

[*] Wilcoxon signed-rank test, $p$-value=$3.20\times10^{-59}$
