# Supplementary material for: Computational Prediction of Heme-Binding Residues by Exploiting Residue Interaction Network
Source: PLoS One. 2011 Oct 3;6(10):e25560. doi: 10.1371/journal.pone.0025560 (PMC3184988; doi:10.1371/journal.pone.0025560)
Supplement: Table S4 — Comparison of the prediction performance for individual holo structure. (PDF) [file pone.0025560.s005.pdf]

Table S4 Comparison of the prediction performance for individual holo structure

Chain	Recall (%)	Precision (%)	Accuracy (%)	F1-score (%)	MCC
1KBI:A	64.52[a] (67.74)[b]	28.99 (26.92)	86.49 (84.91)	40.00 (38.53)	0.370 (0.361)
1N45:A	68.00 (48.00)	47.22 (44.44)	85.86 (85.34)	55.74 (46.15)	0.488 (0.377)
1N5U:A	50.00 (50.00)	11.65 (10.43)	81.47 (79.32)	18.90 (17.27)	0.172 (0.154)
2ITF:A	52.63 (42.11)	52.63 (61.54)	84.35 (86.09)	52.63 (50.00)	0.433 (0.433)
2NWB:A	72.41 (75.86)	38.89 (34.38)	87.94 (85.59)	50.60 (47.31)	0.472 (0.446)
2OFR:X	50.00 (35.71)	50.00 (45.45)	83.63 (82.46)	50.00 (40.00)	0.402 (0.302)
2R7A:A	13.64 (4.55)	42.86 (20.00)	89.73 (88.84)	20.69 (7.41)	0.199 (0.052)
2ZDO:A	88.00 (68.00)	61.11 (65.38)	83.65 (83.65)	72.13 (66.67)	0.631 (0.559)
3CQV:A	62.50 (45.83)	34.88 (33.33)	78.74 (79.89)	44.78 (38.60)	0.350 (0.274)
3EMM:A	56.00 (48.00)	51.85 (52.17)	83.22 (83.22)	53.85 (50.00)	0.437 (0.400)
Average	57.77 (48.58)	42.01 (39.41)	84.51 (83.93)	45.93 (40.19)	0.395 (0.336)
Overall	58.73 (50.00)	35.07 (31.03)	84.65 (83.51)	43.92 (38.30)	0.373 (0.305)

[a] The performance was obtained by HemeNet.

[b] The performance was obtained by the baseline model.
