# Supplementary material for: Computational Prediction of Heme-Binding Residues by Exploiting Residue Interaction Network
Source: PLoS One. 2011 Oct 3;6(10):e25560. doi: 10.1371/journal.pone.0025560 (PMC3184988; doi:10.1371/journal.pone.0025560)
Supplement: Table S5 — Comparison of the prediction performance for individual apo structure. (PDF) [file pone.0025560.s006.pdf]

Table S5 Comparison of the prediction performance for individual apo structure

Chain	Recall (%)	Precision (%)	Accuracy (%)	F1-score (%)	MCC
1SZF:B	45.45[a] (54.55)[b]	14.71 (14.29)	89.55 (87.76)	22.22 (22.64)	0.215 (0.234)
1S8C:D	56.00 (44.00)	51.85 (52.38)	87.43 (87.43)	53.85 (47.83)	0.466 (0.409)
3CX9:A	58.33 (50.00)	15.73 (12.90)	84.66 (83.21)	24.78 (20.51)	0.245 (0.189)
2ITE:B	36.84 (36.84)	53.85 (70.00)	84.35 (86.96)	43.75 (48.28)	0.359 (0.444)
1ZEE:B	73.08 (69.23)	33.33 (30.00)	86.20 (84.66)	45.78 (41.86)	0.431 (0.386)
2OFM:X	50.00 (39.29)	53.85 (52.38)	84.80 (84.21)	51.85 (44.90)	0.429 (0.364)
2RG7:D	31.82 (4.55)	46.67 (12.50)	89.91 (87.72)	37.84 (6.67)	0.333 (0.018)
1XBW:D	65.22 (56.52)	62.50 (68.42)	81.91 (82.98)	63.83 (61.90)	0.518 (0.515)
2V7C:A	37.50 (25.00)	22.22 (18.18)	79.19 (79.87)	27.91 (21.05)	0.174 (0.100)
2A13:A	65.22 (52.17)	51.72 (54.55)	84.51 (85.21)	57.69 (53.33)	0.489 (0.446)
Average	51.95 (43.21)	40.64 (38.56)	85.25 (85.00)	42.95 (36.90)	0.366 (0.311)
Overall	53.46 (43.78)	34.02 (29.87)	85.86 (85.03)	41.58 (35.51)	0.351 (0.280)

[a] The performance was obtained by HemeNet.

[b] The performance was obtained by the baseline model.
